# 878. Association between Anterior Nasal and Plasma SARS-CoV-2 RNA Levels and Hospitalization or Death for Non-Hospitalized Adults with Mild-to-Moderate COVID-19

**DOI:** 10.1093/ofid/ofac492.071

**Published:** 2022-12-15

**Authors:** Mark Giganti, Kara W Chew, Joseph J Eron, Jonathan Z Li, Mauricio Pinilla, Carlee Moser, Arzhang Cyrus Javan, William A Fischer, Paul Klekotka, David A Margolis, David A Wohl, Robert Coombs, Eric S Daar, Davey M Smith, Michael D Hughes, Judith S Currier

**Affiliations:** Harvard T.H. Chan School of Public Health, Boston, MA; David Geffen School of Medicine at University of California, Los Angeles, California; University of North Carolina at Chapel Hill School of Medicine, Chapel Hill, North Carolina; Brigham and Women's Hospital, Boston, MA; Harvard TH Chan School of Public Health, East Boston, Massachusetts; Harvard T.H. Chan School of Public Health, Boston, MA; National Institutes of Health, Bethesda, Maryland; Institute for Global Health and Infectious Diseases, the University of North Carolina, Chapel Hill, North Carolina; Lilly Biotechnology Center, San Diego, California; Brii Biosciences, Durham, NC; University of North Carolina at Chapel Hill School of Medicine, Chapel Hill, North Carolina; University of Washington, Seattle, Washington; Lundquist Institute at Harbor-UCLA Medical Center, Torrance, California; University of California, San Diego, La Jolla, California; Harvard T.H. Chan School of Public Health, Boston, MA; David Geffen School of Medicine at University of California, Los Angeles, California

## Abstract

**Background:**

Data are currently limited on the performance of SARS-CoV-2 RNA levels as predictors or surrogate markers for clinical outcomes in outpatients with mild-to-moderate COVID-19.

**Methods:**

This exploratory analysis used data from 2205 non-hospitalized adults who enrolled between August 2020 and July 2021 and participated in placebo-controlled evaluations of two monoclonal antibody (mAb) agents (bamlanivimab [n=317] or amubarvimab/romlusevimab [n=837]), and an open-label cohort of bamlanivimab recipients [n=1051] as part of the ACTIV-2/A5401 platform trial. SARS-CoV-2 RNA levels were measured in anterior nasal (AN) swabs and plasma at day 0 (pre-treatment) and AN at day 3. We fit regression models to estimate the association between RNA level or detection and subsequent hospitalization/death within 28 days of enrollment.

**Results:**

One-hundred four participants (53/571 [9%] on placebo and 51/1634 [3%] on mAb) died or were hospitalized through day 28. Median AN RNA levels were lower at day 3 compared to day 0 in both placebo (2.5 vs 4.0 log_10_ copies/mL [cp/mL]) and mAb (2.3 vs 4.9) groups. For placebo recipients, higher Day 0 AN RNA was associated with an increasing risk of hospitalization/death, ranging from 3% to 16% for < 2 and ≥ 6 log_10_ cp/mL, respectively. Although only 1% had quantifiable plasma SARS-CoV-2 RNA, there was a similar trend for day 0 plasma RNA: 5% hospitalizations/death for undetectable RNA, 16% for detectable but not quantifiable RNA, and 80% for ≥ 2 log_10_ cp/mL. Among 485 placebo recipients with days 0 and 3 AN RNA results, the risk of subsequent hospitalization/death was highest among those with ≥ 5.0 log_10_ cp/mL at both days [8/78; 10%] and lowest for those with unquantifiable levels at both days [0/124; 0%]. Higher AN RNA at day 3 (adjusted for day 0 RNA) was associated with subsequent hospitalization/death among placebo recipients (relative risk (RR): 1.4 per log_10_ cp/mL; 95%CI: 1.0, 2.1), but not mAb recipients (RR: 1.0; 95%CI: 0.7, 1.6).

**Conclusion:**

These findings suggest that AN and plasma SARS-CoV-2 RNA levels are predictive of hospitalization/death in the natural history setting. However, different associations for mAb and placebo recipients raises concerns for using AN RNA as a surrogate for clinical outcomes in mAb trials.

**Disclosures:**

**Kara W. Chew, M.D., M.S.**, Merck Sharp & Dohme: Grant/Research Support|Pardes Bioscences: Advisor/Consultant **Joseph J. Eron, MD**, GSK: Advisor/Consultant|Merck: Advisor/Consultant **Jonathan Z. Li, MD, MMSc**, Abbvie: Advisor/Consultant|Merck: Grant/Research Support **William A. Fischer, II, MD**, Janssen: Adjudication Committee (Influenza)|Merck: Advisor/Consultant|Ridgeback Biopharmaceuticals: Research funding provided to the University of North Carolina|Roche: Advisor/Consultant|Syneos: Adjudication committee (Influenza) **Paul Klekotka, MD, PhD**, Eli Lilly: Employee|Eli Lilly: Stocks/Bonds **David A. Margolis, MD MPH**, Brii Biosciences: Stocks/Bonds **David A. Wohl, M.D.**, Gilead: Advisor/Consultant|Gilead: Grant/Research Support|Lilly: Grant/Research Support|ViiV: Advisor/Consultant|ViiV: Grant/Research Support **Eric S. Daar, M.D.**, Gilead: Advisor/Consultant|Gilead: Grant/Research Support|Merck: Advisor/Consultant|ViiV: Advisor/Consultant|ViiV: Grant/Research Support **Davey M. Smith, M.D., M.A.S.**, Arena Pharmaceuticals: Advisor/Consultant|Bayer Pharmaceuticals: Advisor/Consultant|Brio Clinical.: Advisor/Consultant|Fluxergy: Advisor/Consultant|Kiadis: Advisor/Consultant|Linear Therapies: Advisor/Consultant|Matrix BioMed: Advisor/Consultant|Model Medicines: Advisor/Consultant|Signant Health: Advisor/Consultant|VxBiosciences: Advisor/Consultant **Judith S. Currier, M.D., MSc**, Merck: Advisor/Consultant.

